# Galactokinase 1 is the source of elevated galactose‐1‐phosphate and cerebrosides are modestly reduced in a mouse model of classic galactosemia

**DOI:** 10.1002/jmd2.12438

**Published:** 2024-06-23

**Authors:** Linley Mangini, Roger Lawrence, Manuel E. Lopez, Timothy C. Graham, Christopher R. Bauer, Hang Nguyen, Cheng Su, John Ramphal, Brett E. Crawford, Tom A. Hartl

**Affiliations:** ^1^ Research and Early Development BioMarin Pharmaceutical Inc. San Rafael California USA

**Keywords:** cerebroside, galactokinase 1 (GALK1), galactose‐1‐phosphate uridylyltransferase (GALT), galactosemia, galactosylceramide, myelin

## Abstract

Classic galactosemia (CG) arises from loss‐of‐function mutations in the *Galt* gene, which codes for the enzyme galactose‐1‐phosphate uridylyltransferase (GALT), a central component in galactose metabolism. The neonatal fatality associated with CG can be prevented by galactose dietary restriction, but for decades it has been known that limiting galactose intake is not a cure and patients often have lasting complications. Even on a low‐galactose diet, GALT's substrate galactose‐1‐phosphate (Gal1P) is elevated and one hypothesis is that elevated Gal1P is a driver of pathology. Here we show that Gal1P levels were elevated above wildtype (WT) in *Galt* mutant mice, while mice doubly mutant for *Galt* and the gene encoding galactokinase 1 *(Galk1)* had normal Gal1P levels. This indicates that GALK1 is necessary for the elevated Gal1P in CG. Another hypothesis to explain the pathology is that an inability to metabolize galactose leads to diminished or disrupted galactosylation of proteins or lipids. Our studies reveal that levels of a subset of cerebrosides—galactosylceramide 24:1, sulfatide 24:1, and glucosylceramide 24:1—were modestly decreased compared to WT. In contrast, gangliosides were unaltered. The observed reduction in these 24:1 cerebrosides may be relevant to the clinical pathology of CG, since the cerebroside galactosylceramide is an important structural component of myelin, the 24:1 species is the most abundant in myelin, and irregularities in white matter, of which myelin is a constituent, have been observed in patients with CG. Therefore, impaired cerebroside production may be a contributing factor to the brain damage that is a common clinical feature of the human disease.

**Synopsis**

An evaluation of biochemical changes in a classic galactosemia (CG) mouse model shows for the first time that galactokinase 1 (GALK1) is the primary source of elevated galactose‐1‐phosphate (Gal1P) in CG; and that cerebroside species known to be important structural components of myelin are reduced, supporting a potential explanation for pathological changes in white matter.

## 1 INTRODUCTION

Classic galactosemia (CG) can manifest as a life‐threatening disorder in neonates, but treatment through dietary restriction of galactose (Gal) resolves the acute complications of the disorder and prevents fatality.^1^ Unfortunately, Gal‐dietary restriction is not a cure, and many patients still experience long‐term complications.^1^ A recent evaluation of The Galactosemia Network's (GalNet's) international registry of CG patients has provided a wealth of information and underscores the residual unmet need for CG patients despite adherence to a Gal‐restrictive diet.^2^ Among their large cohort (*n* = 350), 85% of patients experienced neurological impairments including language and speech disorders (66.4%), tremor (31%), and cognitive developmental delay during infancy/childhood (40%). Primary ovarian insufficiency is a highly penetrant phenotype, occurring in 80% of CG women.^2^ Women with CG can have reduced fertility, although successful pregnancies do happen.^3^ Reduced bone mineral density was also a common finding, occurring in 26.5% of the patients. These findings in the GalNet registry confirmed and extended natural history information collected on CG from several other studies.^4^, ^5^, ^6^, ^7^, ^8^, ^9^, ^10^, ^11^, ^12^

**FIGURE 1:**
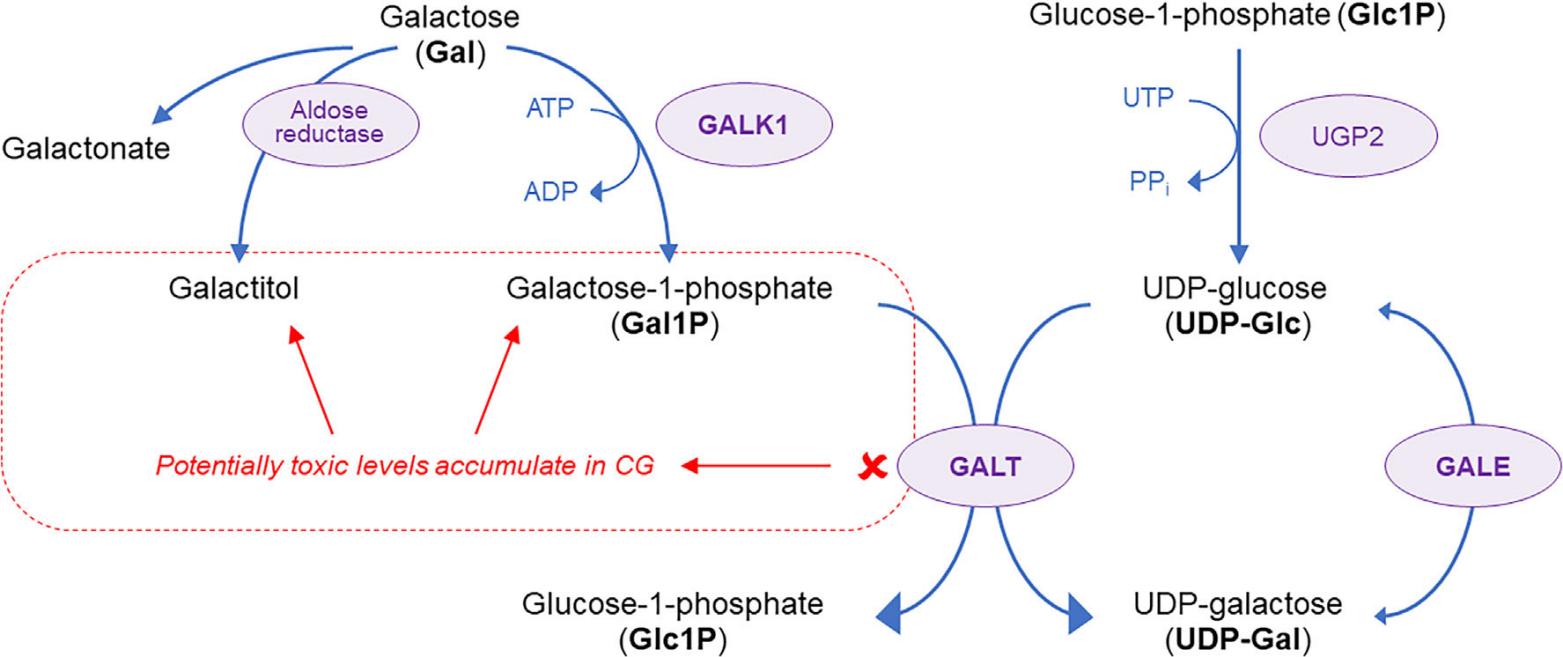
The Leloir pathway of galactose metabolism. ADP, adenosine diphosphate; ATP, adenosine triphosphate; CG, classic galactosemia; GALE, UDP‐galactose‐4′‐epimerase; GALK1, galactokinase 1; GALT, galactose‐1‐phosphate uridylyltransferase; PPi, inorganic pyrophosphate; UDP, uridine diphosphate; UGP2, UDP‐glucose pyrophosphorylase; UTP, uridine triphosphate. *Source*: Adapted from Succoio et al. *Biomolecules*. 2022^13^ and Tang et al. *Anticancer Res*. 2016.^14^

CG is caused by mutations in the *GALT* gene, resulting in a deficiency of the enzyme galactose‐1‐phosphate uridylyltransferase (GALT).^13^ GALT is one of a series of enzymes in the Leloir pathway (Figure 1),^13^, ^14^ which is responsible for Gal metabolism. In the Leloir pathway, dietary or endogenously produced Gal is phosphorylated by galactokinase 1 (GALK1) to produce galactose‐1‐phosphate (Gal1P).^15^, ^16^ Gal1P and uridine diphosphate glucose (UDP‐Glc) are substrates for GALT, which produces glucose‐1‐phosphate (Glc1P) and UDP‐galactose (UDP‐Gal).^17^ UDP‐Gal and UDP‐Glc can be interconverted by the enzyme UDP‐galactose‐4′‐epimerase (GALE).^18^

When GALT is deficient, Gal and Gal1P accumulate at supraphysiological levels.^7^, ^19^, ^20^ Galactonate and the sugar alcohol galactitol, which is formed from Gal via the enzyme aldose reductase, also accumulate to high levels when Gal is elevated (Figure 1).^13^, ^20^, ^21^, ^22^ Since removal of Gal from the diet of CG newborns provides substantial benefit, it is likely that Gal and/or one or more of its metabolites (Gal1P and/or galactitol) are toxic during the neonatal period.

The mechanisms by which accumulation of Gal and/or its metabolites exert toxicity in CG are uncertain but there are several possibilities. The act of elevated synthesis of Gal1P and/or galactitol may consume other critical metabolites. Elevated Gal1P synthesis has been shown to deplete cellular ATP levels^23^, ^24^ and galactitol synthesis consumes NADPH. Since NADPH is a cofactor for glutathione reductase, its depletion may explain the signatures of oxidative stress that have been observed in CG animal models.^25^, ^26^, ^27^, ^28^ In addition, high levels of Gal1P and/or galactitol may themselves directly drive toxicity. Gal and/or its metabolites have been shown in vitro to inhibit a suite of other saccharide regulatory enzymes likely by binding within their active sites and reducing substrate binding.^24^, ^25^ High levels of intracellular galactitol disturbs osmotic balance and has been proposed to explain the commonly occurring cataracts phenotype.^29^, ^30^

It is not understood why CG patients suffer long‐term complications despite being on life‐long Gal dietary restriction. There are three favored and non‐mutually exclusive hypotheses. One is that pathology is induced prior to Gal dietary restriction, for example, in utero or in the period prior to diagnosis and initiation of Gal‐dietary restriction. Such pathology, if permanent, could lead to long‐term clinical manifestations. The second hypothesis is that Gal and its metabolites, which remain elevated even on Gal‐dietary restriction because of endogenous Gal production,^7^, ^19^, ^20^, ^21^, ^22^, ^31^ may continue to reach toxic levels, and thus may continue to cause damage. A third, less favored hypothesis is that the “break” in the Leloir pathway leads to a deficiency in the product UDP‐Gal; however, there are contradictory reports on UDP‐Gal levels in cellular and animal models of CG – in some instances UDP‐Gal levels were lower in GALT‐deficient models than in wildtype (WT),^32^, ^33^, ^34^, ^35^, ^36^, ^37^ whereas in other cases there was no difference.^38^, ^39^, ^40^ Furthermore, UDP‐Gal production is not solely dependent on GALT since UDP‐Gal can be generated from UDP‐Glc via the enzyme GALE, and UDP‐Glc is produced from Glc1P and UTP via the ubiquitous enzyme UDP‐glucose pyrophosphorylase (UGP2) (Figure 1). Nevertheless, there may be circumstances when the demand for UDP‐Gal is high, thereby making CG patients especially susceptible.

Numerous studies have shown irregular glycosylation of proteins and/or lipids in CG patient tissues,^41^, ^42^ serum^43^, ^44^, ^45^, ^46^, ^47^, ^48^, ^49^, ^50^ and cell lines.^51^, ^52^ Improper glycosylation could lead to protein instability and explain the endoplasmic reticulum stress and unfolded protein response observed in GALT‐deficient models.^53^, ^54^, ^55^ As discussed previously,^56^, ^57^ irregular glycosylation has been theorized to occur either via a deficiency in the Gal donor UDP‐Gal^58^ or as the result of diminished activity of the glycosyltransferases that donate hexoses into growing glycan chains using UDP‐hexoses as substrates.^56^ CG is considered a secondary congenital disorder of glycosylation (CDG), and CG patients can have altered transferrin mobility in diagnostic tests because the transferrin protein is improperly glycosylated.^43^, ^49^, ^59^ Disrupted glycosylation is an attractive mechanism of disease pathology because CG patients display irregular white matter,^41^, ^60^, ^61^, ^62^, ^63^, ^64^, ^65^, ^66^, ^67^, ^68^, ^69^, ^70^ which is rich in galactose‐bearing lipids. Indeed, approximately 25% of myelin's lipid content is made up of the cerebroside galactosylceramide (GalCer; ~20%) and its 3‐O sulfated derivatives (sulfatides; ~5%),^71^ which are necessary for the stability and maintenance of myelin.^72^, ^73^, ^74^ Autopsies of galactosemic infants have shown decreased levels of cerebrosides in the brain as compared to age‐matched controls.^41^, ^42^

The present study aimed to determine whether GALK1 is the primary source of elevated Gal1P when *Galt* is mutant. We show for the first time that GALK1 is the primary source of elevated Gal1P in the liver and brain of GALT‐deficient mice, as *Galk1/Galt* double mutant mice demonstrated WT levels of Gal1P in those tissues. This argues that the paralog GALK2 is likely not a significant contributor to liver and brain Gal1P elevations in GALT‐deficiency. We also set out to determine if brain sphingolipids are altered in mice mutant for *Galt*. In *Galt‐*mutant mice, we observed modest, yet significant, reduction of the 24:1 cerebrosides, including GalCer 24:1, which is the most abundant cerebroside species in myelin.^74^, ^75^ In contrast, no significant changes to a variety of gangliosides were detected. Cerebroside reduction is consistent with white matter abnormalities that have been previously documented in CG patients^41^, ^60^, ^61^, ^62^, ^63^, ^64^, ^65^, ^66^, ^67^, ^68^, ^69^, ^70^ including two patient autopsies where cerebroside levels were found to be depleted.^41^, ^42^

## 2 MATERIALS AND METHODS

### 2.1 Animals

The 9‐, 11‐, and 36‐week‐old *Galt* ^−/−^ and sibling WT animals were housed, and the experimental work was performed, in an American Association for Laboratory Animal Science (AALAS)‐accredited vivarium facility at the Buck Institute for Research on Aging, Novato, CA, USA. These mice were fed 2018S Teklad Global 18% Protein Rodent Diet by Envigo (Indianapolis, IN, USA). All work was approved by the Buck IACUC committee protocol #A10197. Animals were euthanized either by controlled CO_2_ inhalation or cardiac puncture and thoracotomy while under surgical plane anesthesia.

The 3‐week‐old WT, *Galt* ^−/−^, *Galk1* ^−/−^, *Galt* ^−/−^/*Galk1* ^+/−^, and *Galt* ^−/−^/*Galk1* ^−/−^ siblings (*n* = 5 per group) were bred, maintained in pressurized intra‐ventilated caging and the study was conducted by In Vivo Services at The Jackson Laboratory (Sacramento, CA, USA). These mice were fed LabDiet® JL Rat and Mouse/Auto 6F 5K52 chow (LabDiet, Richmond, IN, USA). All work was performed under Institutional Animal Care and Use Committee (IACUC)‐reviewed and approved protocols. The Jackson Laboratory is an Office of Laboratory Animal Welfare (OLAW)‐assured and Association for Assessment and Accreditation of Laboratory Animal Care International (AAALACi)‐accredited organization. Mice were regularly observed for any signs of abnormalities. Any mice suspected of being ill or injured were immediately reported to veterinary staff and treated as directed by the veterinary staff. Euthanasia was performed using compressed CO_2_ gas, according to American Veterinary Medical Association (AVMA) guidelines.

The *Galk* ^−/−^ strain was kindly provided by Dr. Dwight Stambolian (University of Pennsylvania School of Medicine).^30^

#### 2.1.1 *Galt*‐mutant mouse generation

The *Galt* knockout (KO) mice were generated at The Jackson Laboratory, with all work completed at the AAALAC‐accredited site at Bar Harbor, ME, USA. This work was carried out in strict accordance with the recommendations in the Guide for the Care and Use of Laboratory Animals of the National Institutes of Health. The protocol was approved by the IACUC at The Jackson Laboratory (Protocol Number 10010 for Genetic Engineering Technologies).

**FIGURE 2:**
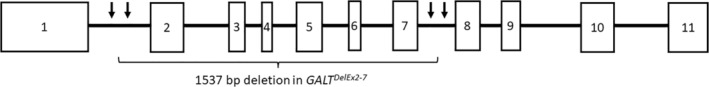
The *Galt* ^*DelEx2*^ ^–*7*^ allele. sgRNAs were designed to target introns 1 and 7 (arrows). A 1537‐bp deletion was generated that removes exons 2 to 7 and parts of introns 1 and 7. Gene structure modeled after ENSMUST00000084695.11.

To generate mice with an intragenic deletion of the *Galt* gene, two single guide RNAs (sgRNAs) targeting the intron 1 sequences (CCCCTCCGCCAGCCTATAGCTTC and TAGGGCATTAGAGCCACTCGGG) and two targeting the intron 7 sequences (ATATTATGGTGGCTTAGGGTAGG and CCCTCTCACATGCACGATTCACT) were designed to recruit Cas9 to these two genomic regions. Double‐strand breaks at these regions, followed by a non‐homologous end joining event, would produce an intragenic deletion that spans exons 2 to 7. Paired sgRNAs were electroporated at 600 ng/μL each along with spCas9 mRNA at 500 ng/μL in a volume of 10 μL added to 10 μL of Opti‐MEM media with zygotes from C57BL/6J mice (JAX #000664, The Jackson Laboratory, Bar Harbor, ME, USA). The electroporated zygotes were implanted into pseudopregnant fosters. A male founder with a 1537‐bp deletion that removes exons 2–7 and parts of introns 1 and 7 (Figure 2) was identified and bred to C57BL/6J mice. The deletion removes bps 41 755 833–41 757 369 on chromosome 4 in GRCm39 (NC_000070.7) and consequently exons 2–7 of the *Galt* gene, and includes the nucleotides coding for the enzyme's His‐Pro‐His active site.^76^, ^77^, ^78^ It is similar to the allele in the original *Galt* KO mouse, which was a deletion from exons 6 to 8 and had no detectable *Galt* enzymatic activity.^79^ It is likely that the *Galt* ^*DelEx2*–*7*^ allele is non‐functional.

#### 2.1.2 Western blot analysis

A small piece of liver (~100 μg) was homogenized in RIPA buffer with Halt™ Protease Inhibitor and Pierce™ Universal Nuclease (all by Thermo Fisher Scientific) at a concentration of 5 μL buffer per 1 μg tissue. Samples were homogenized using an Omni Bead Ruptor 24 Homogenizer following the manufacturer's protocol (Omni International, Kennesaw, GA, USA) and then centrifuged. The supernatant was saved and its protein concentration was determined using the BCA assay. All homogenates were diluted with RIPA buffer to a concentration of 4 μg protein/μL.

For each sample, 20 μg of protein was reduced and denatured with Thermo Fisher NuPage LDS Sample Buffer and Reducing Agent. These samples, alongside SeeBlue Plus2 Pre‐stained Protein Standard Ladder, were loaded onto a 4%–12% Bis‐Tris gel, and gels were electrophoresed for 70 min at 150 V in MES buffer. Proteins were then transferred onto a nitrocellulose blot using the Thermo Fisher iBlot 2 Transfer system.

Blots were first blocked overnight and then incubated with an anti‐GALT antibody at 1:2000 overnight at 4°C (Abcam, ab178406). Blots were then washed and incubated with a secondary anti‐rabbit IgG HRP‐conjugated antibody at 1:3000 for 1 h at room temperature (Promega, W4018). Following secondary antibody incubation, blots were washed, incubated with Immobilon Crescendo Western HRP Substrate (Millipore), and imaged for chemiluminescence using an Azure Biosystems c400 imager (Dublin, CA, USA).

After GALT detection, blots were stripped using Restore Plus Western Blot Stripping Buffer, and then re‐blocked overnight. Blots were then incubated with an anti‐GAPDH peroxidase‐conjugated antibody at 1:30 000 for 1 h at room temperature (Sigma‐Aldrich, G9295). Following antibody incubation, blots were washed, incubated with Immobilon Crescendo Western HRP Substrate (Millipore), and imaged using an Azure Biosystems c400 imager.

### 2.2 Gal1P and sphingolipid analyses

#### 2.2.1 Sample homogenate preparation

Approximately 200 mg of each brain or liver tissue sample was homogenized in 1 mL of water with 1.4 mm ceramic beads using an Omni Bead Ruptor 24 Homogenizer following the manufacturer's protocol (Omni International, Kennesaw, GA, USA). Protein concentration of the homogenate was determined using the standard BCA assay. All homogenates were diluted with water to a concentration of 4 μg protein/μL.

#### 2.2.2 Gal1P sample preparation and derivatization

Gal1P standard curve and Gal1P‐containing biological samples were derivatized as previously described.^80^ Thirty microliters of the brain or liver homogenate was extracted with 100 μL of methanol containing 1 μg/mL (^13^C_6_) Gal1P as an internal standard. After centrifugation, 85 μL of the clarified supernatant was dried down and subjected to a two‐step derivatization process that differentiates between Gal1P and its isobaric isomer Gal6P. The dried pellets were reconstituted into 40 μL of methoxyamination reagent (25 mg/mL methoxylamine hydrochloride in anhydrous pyridine) and incubated for 1 h at 60°C. This step forms methoxime derivatives of free carbonyl‐containing sugars such as Gal6P while leaving sugars without a free carbonyl, such as Gal1P, unmodified.^80^ The free hydroxyl groups in both methoxime derivatized and underivatized sugars were subsequently esterified by adding 12 μL of propionic anhydride and 24 μL of 1‐methylimidazole and incubating for 1 h at 60°C. Finally, 150 μL of acetonitrile was added and the samples were briefly vortexed and then centrifuged. For the LC–MS/MS analysis, 50 μL of sample was mixed with an equal volume of water and 4 μL of the diluted sample was analyzed. Under these chromatographic conditions, the elution time for Gal1P is 8.4 min, which differs from those of the isobaric species Glc1P and Man1P. For Gal6P, Glc6P, Man6P, Fru6P, and Fru1P, this derivatization scheme yields products with a mass that differs from derivatized Gal1P, Glc1P, and Man1P. These differences allowed a distinction to be made between Gal1P and other phosphosugars for accurate quantitation.

#### 2.2.3 Gal1P liquid chromatography–mass spectrometry analysis

LC–MS/MS analysis was performed on a Shimadzu UHPLC system (Shimadzu Corporation, Kyoto, Japan) equipped with an Acquity UPLC HSS T3 column (2.1 mm × 50 mm, 1.8 μm) (Waters, Milford, MA, USA) connected to a Sciex API 4000 Triple Quad mass Spectrometer (Framingham, MA, USA). Solvent A was made in water with 0.125% formic acid and 2 mM ammonium acetate and containing 0.2% HFIP (1,1,1,3,3,3‐Hexafluoro‐2‐propanol, Sigma‐Aldrich) as an acidic modifier; solvent B was acetonitrile containing 0.1% formic acid. The initial composition was 95% A/5% B at a flow rate of 0.5 mL/min with the column temperature kept at 50°C. For the Gal1P analysis, 4 μL of the derivatized sample was injected and elution was carried out using a gradient method of 95% A/5% B to 85% A/15% B over the first 2 min; 85% A/15% B to 65% A/35% B from 2 to 15 min; 65% A/35% B to 5% A/95% from 15 to 16 min; 5% A/95% B isocratic from 16 to 18 min; 5% A/95% B to 95% A/5% B from 18 to 18.5 min; and then re‐equilibration to 20 min.

The API 4000 was operated in the positive ion mode and the sample ionized by ESI. The ion source temperature was set at 600°C with an ion spray voltage of 5.5 kV, curtain gas at 20 and collision gas at 5 arbitrary units. Multiple reaction monitoring was used to quantify Gal1P using an eight‐point standard curve (10–10 000 ng/mL). Results were normalized to the heavy Gal1P internal standard. Monitored transitions and parameters used in LC–MS/MS detection of Gal1P are shown in Table 1.

**TABLE 1:** Monitored transitions and individual parameters used in LC–MS/MS detection of galactose‐1‐phosphate (Gal1P).

Analyte	Precursor ion	Product ion	Collision energy	Q2 exit potential
Gal1P	466.3	387.3	13	15
Gal1P	466.3	183.2	22	15
Gal1P	466.3	109.2	26	15
Gal1P	466.3	127.2	36	15
Gal1P	466.3	239.2	19	15
Gal1P	466.3	81.2	73	15
(^13^C_6_)Gal1P	472.4	393.3	13	15
(^13^C_6_)Gal1P	472.4	189.1	22	15

#### 2.2.4 Cerebroside analysis

Brain tissue samples were extracted according to a method adapted from Sidhu et al.^81^ Briefly, 50 μL of the sample homogenate (equal to 200 μg of protein) was extracted with 10 μL of 0.5 N sodium hydroxide in water and vortexed briefly. Then 10 μL of an internal standard mix and 920 μL of acetonitrile were added to the sample, and samples were vortexed again. Finally, 10 μL of 10% formic acid in acetonitrile was added to the samples and vortexed briefly. Samples were then centrifuged, and the supernatants were transferred to new tubes and stored at −20°C until use. Samples were analyzed for glycosylated‐sphingosines and ‐ceramides on an Acquity ultra‐high‐performance liquid chromatography (UPLC) attached to a Xevo TQ‐S micro Triple Quadrupole Mass Spectrometer (Waters Corporation, Milford, MA).

Cerebroside standards were purchased from Avanti Polar Lipids (Alabaster, AL, USA). All GalCer, sulfatide and C20 and C22 glucosylceramide (GlcCer) species were quantified relatively, utilizing the standard curves generated for the GlcCer species with the same length fatty acid chain or the GlcCer form with the most similar retention time (e.g., C16 GalCer was quantitated using the C16 GlcCer standard curve). All stock standards were prepared in 95/5 methanol/glacial acetic acid. The internal, working and calibration standards were prepared in acetonitrile unless otherwise specified. The internal standard was prepared as a mixture of glucosyl sphingosine‐d5, galactosyl sphingosine‐d5, and C18 GlcCer‐d5 at 2.5 ng/μL. Working standards were prepared as a mixture of glucosyl sphingosine, C16 GlcCer, C18 GlcCer, and C24:1 GlcCer at concentrations ranging from 10 ng/μL to 1 pg/μL. The 10 ng/μL working standard had a final solvent mix of 50% 95/5 methanol/glacial acetic acid and 50% acetonitrile due to solubility problems in acetonitrile at that highest concentration. The working standards were then used to prepare an external standard curve with the internal standard for all the compounds in a similar manner to that used for sample extraction, except that 50 μL of water was substituted for sample homogenate, and the working standard was substituted for a portion of the acetonitrile added.

The glycolipid analytical method was adapted from Boutin et al.^82^ Glycolipids were separated on a Halo HILIC column (Advanced Materials Technology, Wilmington, DE, USA). Solvent A was 5 mM ammonium formate in 97.5% acetonitrile, 2% water, and 0.5% formic acid. Solvent B was 5 mM ammonium formate in 50% methanol, 49.5% water, and 0.5% formic acid. The initial solvent composition was 100% A at a flow rate of 0.4 mL/min. The column was kept at ambient temperature. The LC elution gradient profile was as follows: hold at 100% A for 3 min, ramp to 90% A/10% B over 0.25 min, hold at 90% A/10% B for 6 min, switch to 20% A/80% B and hold for 2.5 min, and finally return to the initial condition of 100% A and re‐equilibrate for 2.5 min. The samples were ionized by ESI in positive ion mode. The capillary voltage was set at 3.0 kV, the desolvation temperature was 350°C and the desolvation gas flow speed was 650 L/hr. One precursor‐product ion transition was monitored for each of the glycosphingolipid species as follows: glycosyl sphingosine‐d5, 467.3 > 287.3; glycosyl sphingosine, 462.3 > 282.3; C18 glycosyl ceramide‐d5, 733.5 > 269.3; C16 glycosyl ceramide, 700.5 > 264.3; C18 glycosyl ceramide, 728.5 > 264.3; C24:1 glycosyl ceramide, 810.6 > 264.3. Glucosylated and galactosylated forms of each species shared the same precursor‐product ion transition, but were well resolved, allowing for identification based on retention time.

The results were aggregated across the experiments by first converting each metabolite measurement to “% of normal.” For each group (3‐week‐old males, 9‐week‐old males, 11‐week‐old females, and 39‐week‐old females) normal was defined as the mean cerebroside level of WT mice of the same age. Individual measurements were then normalized to this value and expressed as a percentage.

#### 2.2.5 Ganglioside analysis

Fifty microliters of brain homogenate (equal to 200 μg of protein) was extracted with 95/5 methanol/glacial acetic acid (v/v). Samples were filtered through 10 kDa centrifugal filter tubes and stored at −20°C until use. Brain tissue samples were analyzed for five different gangliosides (GM1, GM2, GM3, GA1, GA2) with an Acquity UPLC system attached to a Xevo TQ‐S micro Triple Quadrupole Mass Spectrometer (Waters Corporation, Milford, MA, USA).

Gangliosides were separated on an Acquity UPLC Glycan BEH Amide column (Waters Corporation, Milford, MA, USA). Solvent A was 5 mM ammonium acetate in 94.5% acetonitrile, 2.5% methanol, 2.5% water, and 0.5% formic acid. Solvent B was water. The initial solvent composition was 95% A/5% B at a flow rate of 0.4 mL/min. The column was kept at 50°C. The LC elution gradient profile was as follows: hold at 95% A/5% B for 2 min, ramp to 50% A/50% B over 10 min, hold at 50% A/50% B for 10 min, then back to 95% A/5% B and hold for 4 min. Samples were ionized by electrospray ionization (ESI) in positive ion mode. The capillary voltage was set at 1.0 kV, the desolvation temperature was 500°C, and the desolvation gas flow speed was 1000 L/h. Two precursor‐product ion transitions, one for the (d18:1/18:0) species, one for the (d18:1/20:0) species, were monitored for each of the five gangliosides at the corresponding mass‐to‐charge ranges as follows: GM1–1546.7 > 366.1, 1574.7 > 366.1; GA1–1255.7 > 366.1, 1283.8 > 366.1; GM2–1384.7 > 204.1, 1412.7 > 204.1; GA2–1093.6 > 264.3, 1121.6 > 292.3; GM3–1181.5 > 264.3, 1209.6 > 292.3. The sum of the two transitions for each ganglioside was used for quantitation. A standard reference curve containing all five gangliosides across a concentration range 100–6.25 pg/μL was prepared using standards from Enzo Life Sciences, Inc. (Farmingdale, NY, USA) in 95/5 v/v methanol/glacial acetic acid.

### 2.3 Statistical analyses

Two‐way ANOVA (analysis of variance) was conducted for each cerebroside to assess the differences between WT and *Galt* ^−/−^ mice, across the mice age groups and within each age group. Data transformation was determined by Box‐Cox transformation when needed. Multiple comparison adjustment for *p*‐values was conducted using the Hommel method, separately for *p*‐values on primary cerebrosides of interest (GalCer 24:1, sulfatide 24:1, and GlcCer 24:1) and secondary cerebrosides of interest (GalCer 16:0, GalCer 18:0, GalCer 20:0, GalCer 22:0, sulfatide 18:0, sulfatide 18:0 2R‐OH, GlcCer 16:0, GlcCer 18:0, GlcCer 20:0, and GlcCer 22:0). All statistical analyses were conducted using R 4.3.2.

## 3 RESULTS

### 3.1 Gal1P is elevated in GALT‐deficient mice yet is normal in GALT/GALK1 double‐deficiency

**FIGURE 3:**
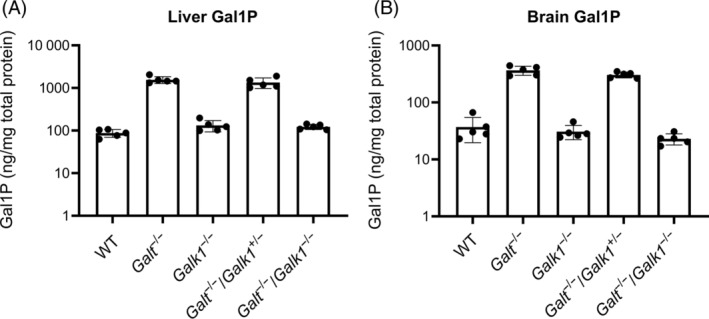
Gal1P levels in *Galt*, *Galk1*, and *Galt*/*Galk1* double mutant mice. Gal1P was measured in liver (A) and brain (B) homogenates from five groups of 3‐week‐old mice: WT, *Galt* ^−/−^, *Galk1* ^−/−^, *Galt* ^−/−^/*Galk1* ^+/−^, and *Galt* ^−/−^/*Galk1* ^−/−^; *n* = 5 per group). In both tissues, Gal1P levels were ~10 to 20‐fold higher in the *Galt* ^*DelEx2*^ ^–*7*^ homozygous (*Galt* ^−/−^) tissues compared to those measured in WT sibling tissues. Introduction of one copy of the *Galk1* ^*tm1Stb*^ null allele did not impact the high levels of Gal1P in the *Galt* ^*DelEx2*^ ^–*7*^ homozygotes (*Galt* ^−/−^/*Galk* ^+/−^), but having two copies of the *Galk1* ^*tm1Stb*^ null allele in the *Galt* ^*DelEx2*^ ^–*7*^ homozygotes (*Galt* ^−/−^/*Galk* ^−/−^) decreased Gal1P levels to those of the WT mice. Data shown are mean values ± SD; dots denote data values for all individual animals included in the experiment. Gal1P, galactose‐1‐phosphate; SD, standard deviation; WT, wildtype.

Gal1P accumulates in CG patients because of the deficiency of GALT (Figure 1). To evaluate Gal1P levels in mice confirmed by western blot to be GALT‐deficient (Figure S1), an LC–MS method that enables analytical distinguishing of Gal1P from other phospho‐hexoses^80^ was implemented. Mean (± standard deviation [SD]) Gal1P levels in the 3‐week‐old WT male mouse liver and brain tissues were 87.6 ± 18.2 ng/mg and 37.1 ± 17.4 ng/mg total protein, respectively. Male siblings of the same age that were *Galt* ^*DelEx2*–*7*^ homozygotes (*Galt* ^−/−^) showed Gal1P levels that were ~10–20× higher than those in WT animals: 1560.1 ± 282.6 ng/mg total protein (liver; Figure 3A) and 366.0 ± 66.2 ng/mg total protein (brain; Figure 3B and Table S1). Notably, this fold‐change in Gal1P levels between WT and *Galt* mutant mice is similar to those measured previously when the original *Galt* KO mice were compared to their WT littermates at 7 weeks of age.^39^ Introduction of a single *Galk1* KO allele^30^ to *Galt* ^−/−^ mice (*Galt* ^*DelEx2*–*7*^/*Galk1* ^*tm1Stb*/+^; *Galt* ^−/−^/*Galk1* ^+/−^) had no impact on the high levels of Gal1P from *Galt* loss as they were equivalent to those from the *Galt* ^−/−^ mice (Figure 3A,B, and Table S1). However, homozygous deficiency of *Galk1* in *Galt* ^−/−^ mice (*Galt* ^−/−^/*Galk1* ^−/−^) led to significant reductions in Gal1P relative to *Galt* ^−/−^ mice (Figure 3A,B). GALK1 is the primary enzyme that phosphorylates Gal^16^ (Figure 1). A paralogous enzyme, GALK2, can phosphorylate Gal in vitro, though inefficiently with a Km ≈ 4 mM.^83^ Since Gal accumulates in *Galt* KO mice,^39^ GALK2 in theory could produce Gal1P when GALT is deficient. However, since the *Galt* ^−/−^/*Galk1* ^−/−^ mice have strong reductions in Gal1P relative to Galt^−/−^ mice, GALK2 has little‐to‐no contribution to the elevated Gal1P in the liver and brain tissues of the GALT‐deficient mice. Indeed, the *Galk1* KO mouse (*Galk1* ^−/−^) very poorly metabolizes Gal,^30^ providing further support that GALK2 cannot fully compensate for loss of *Galk1*.

### 3.2 Reductions in brain 24:1 cerebrosides of GALT‐deficient mice

In addition to being a product of GALT (Figure 1), UDP‐Gal is a substrate for ceramide galactosyltransferase (UGT8), the enzyme that synthesizes the cerebroside GalCer. Approximately 20% of the lipid content of myelin is GalCer and a further 5% is the 3‐O sulfated derivative of GalCer, sulfatide.^71^ The 24:1 acyl chain species of GalCer is the most prominent cerebroside in myelin.^75^ Cerebrosides are necessary for the structural integrity and maintenance of myelin.^72^, ^73^, ^74^ Since myelin‐rich white matter is disrupted in CG patients^41^, ^60^, ^61^, ^62^, ^63^, ^64^, ^65^, ^66^, ^67^, ^68^, ^69^, ^70^ and an autopsied brain from a CG patient had reduced GalCer,^42^ we hypothesized that the most myelin‐enriched cerebroside, GalCer 24:1, and its sulfated derivative, sulfatide 24:1, may be impacted in the GALT‐deficient mouse brain.

**FIGURE 4:**
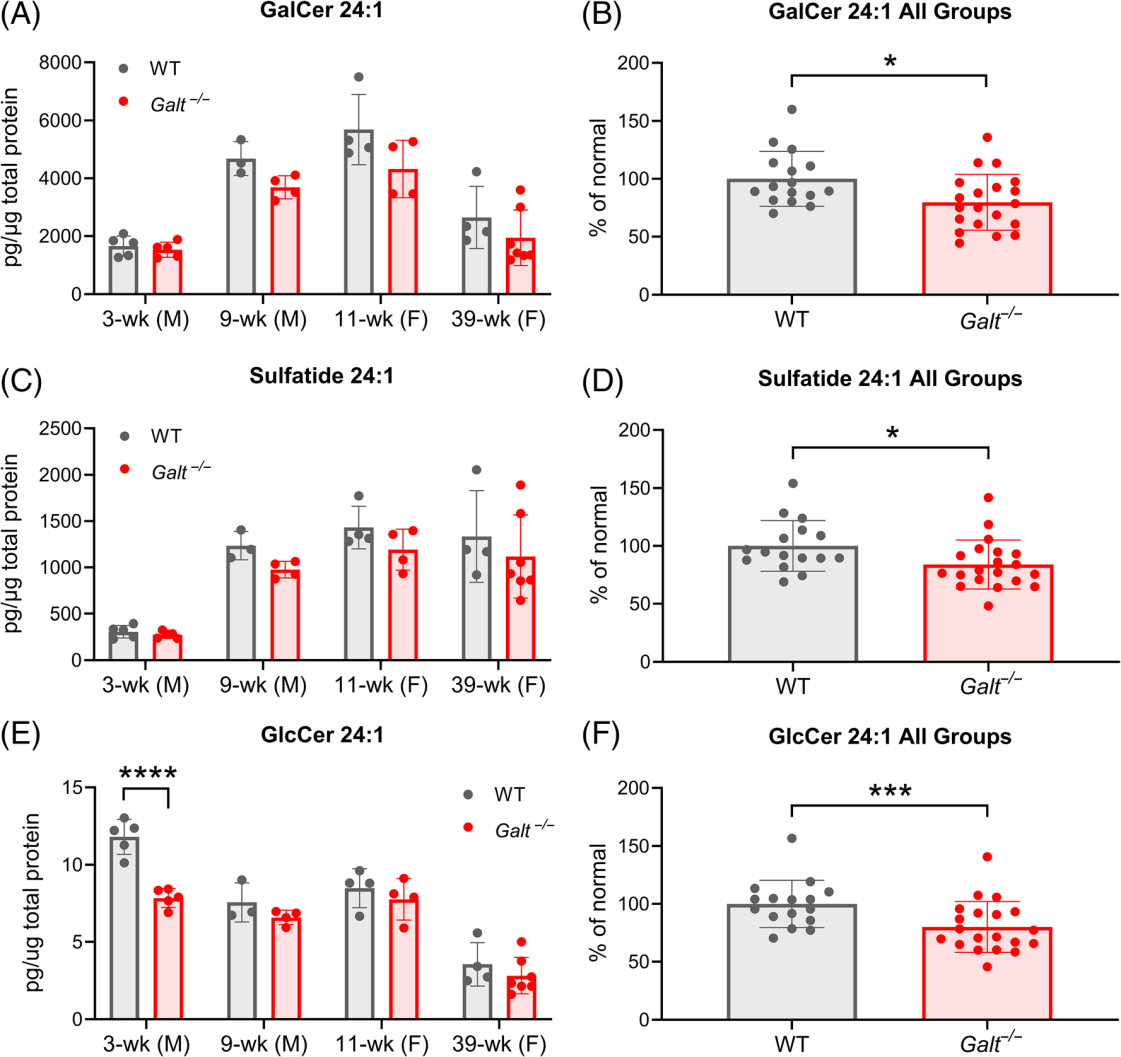
Reduced 24:1 cerebrosides in the brains of the GALT‐deficient mice. Brain levels of cerebrosides were measured by LC–MS in groups of WT and *Galt* ^−/−^ mice euthanized at 3 weeks of age (*n* = 5 per group; all male), 9 weeks (*n* = 3 WT, *n* = 4 *Galt* ^−/−^; all male), 11 weeks (*n* = 4 per group; all female), and 39 weeks (*n* = 4 WT, *n* = 7 *Galt* ^−/−^; all female). (A) GalCer 24:1 trended toward reduction in all age groups but none met statistical significance. (B) For all age groups combined (*n* = 16 WT, *n* = 20 *Galt* ^−/−^), GalCer 24:1 amounts expressed as a percentage of WT levels were reduced in the GALT‐deficient animals by 20.3% (*p* = 0.0421; two‐way ANOVA with Hommel method for multiple comparison). (C) Sulfatide 24:1 trended toward reduction in all age groups but none met statistical significance. (D) For all age groups combined, sulfatide 24:1 amounts expressed as a percentage of WT levels were reduced in the GALT‐deficient animals by 16.0% (*p* = 0.0443; two‐way ANOVA with Hommel method for multiple comparison). (E) GlcCer 24:1 was reduced by 33.6% in the 3‐week‐old GALT‐deficient mice compared with WT (*p* = 0.0001) and trended toward reduction in the other three age groups. (F) For all age groups combined, GlcCer 24:1 amounts expressed as a percentage of WT levels were reduced in the GALT‐deficient animals by 19.9% (*p* = 0.0003; two‐way ANOVA with Hommel method for multiple comparison). Data shown are mean values ± SD; dots denote data values for all individual animals included in the experiment. *p < 0.05. F, female; GalCer, galactosylceramide; GlcCer, glucosylceramide; M, male; SD, standard deviation; wk, week; WT, wildtype.

Levels of GalCer 24:1, sulfatide 24:1, and additionally GlcCer 24:1 from four different groups of animals were evaluated in this study: 3‐week‐old males, 9‐week‐old males, 11‐week‐old females, and 39‐week‐old females (Tables S2–S5 and S10). For each group, 3–7 WT and GALT‐deficient littermates were evaluated (mean [± SD] 4.5 ± 1.2 animals per group). While no overt changes in GalCer 24:1 or sulfatide 24:1 were observed in any individual age group, trends suggested a subtle reduction in the GALT‐deficient animals that were not statistically significant (Figure 4A,C). However, in the 3‐week‐old males, GlcCer 24:1 was reduced by 33.6% (*p* = 0.0001; Figure 4E). When all age groups were combined by expressing cerebroside levels as a percentage of the WT values, GalCer 24:1 was reduced on average by 20.3%, sulfatide 24:1 by 16.0%, and GlcCer 24:1 by 19.9% (*p* = 0.0421, 0.0443, and 0.0003, respectively; Figure 4B,D,F).

Levels of 10 other cerebroside species were analyzed from the same four groups of animals. In total, 7 of 10 species demonstrated a trend toward reduction in the brains of the GALT‐deficient animals, though none of those reductions were statistically significant (Figure S2). All cerebroside measurements are presented in Tables S2–S5 and S10.

Notably, while no specific behavioral studies were conducted, upon general observation, no obvious behavioral abnormalities were observed in *Galt* ^−/−^ mice including seven female mice up to 39 weeks in age. This demonstrates that any reductions in cerebrosides or other biochemical changes are not profound enough to result in overt behavioral changes such as the tremors and weakness existing in *UGT8* mice, which cannot synthesize GalCer or sulfatides.^72^, ^73^ Since CG patients often have clear neurological impairments, these data suggest that our GALT‐deficient mouse may not fully represent the biochemical etiology and/or clinical manifestations of CG in humans.

### 3.3 Ganglioside levels are unaltered in the brains of GALT‐deficient mice

**FIGURE 5:**
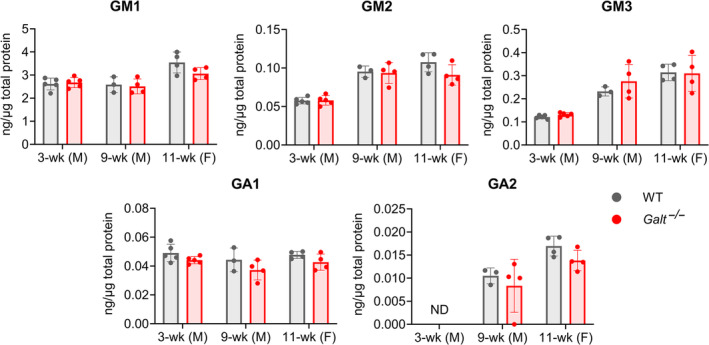
Ganglioside levels are unchanged in the brains of GALT‐deficient mice. Five species of gangliosides were measured via LC–MS from the brains of the WT and the GALT‐deficient 3‐week‐old male, 9‐week‐old male, and 11‐week‐old female mice. No statistically significant decrease in any ganglioside was observed in any of the three groups. Data shown are mean values ± SD; dots denote data values for all individual animals included in the experiment. ND, not detectable; SD, standard deviation; wk, week; WT, wildtype.

Alterations in ganglioside levels have been reported in a brain autopsy from a CG patient, where gangliosides GM3 and GA2 were at levels 706% and 12% of normal, respectively.^42^ In the present study, brain levels of gangliosides GM1, GM2, GM3, GA1, and GA2 measured in the 3‐week‐old males, 9‐week‐old males, and 11‐week‐old females were not significantly different in the *Galt* ^−/−^ mice compared with the WT littermates (Figure 5; Tables S6–S8). Since these five gangliosides are derived from GlcCer,^74^ these findings suggest that changes observed in GlcCer (Figure 4) were not sufficiently profound to impact ganglioside levels.

## 4 DISCUSSION

### 4.1 Elevated Gal1P levels in the *Galt* ^−/−^ mouse and their normalization in the *Galt*/*Galk1* double mutant

As expected, Gal1P levels were elevated in the liver and brain tissues of our *Galt* ^−/−^ (*Galt* ^*DelEx2*–*7*^) mice versus WT siblings. Specifically, Gal1P was elevated by 17.8‐fold in the liver and 9.9‐fold in the brain (Figure 3A,B and Table S1). These Gal1P levels are elevated over WT to a similar extent as what was reported previously in the original *Galt* KO mouse: liver, 1.4 ± 0.16 versus 0.11 ± 0.02 μmol/g (a 12.7‐fold elevation); brain, 0.22 ± 0.02 versus 0.02 ± 0.00 μmol/g (a 10‐fold elevation).^39^ This argues that the newly generated *Galt*‐mutant mouse model used in the present study is also null. Further support for null behavior comes from the large deletion size, which removes the sequence encoding the catalytic domain, and the absence of detectable GALT protein in liver by western analysis (Figure S1).

Crossing two copies of a null *Galk1* allele into the *Galt* ^*DelEx2*–*7*^ background normalized Gal1P levels (Figure 3). This epistatic relationship has been reported previously in yeast^84^ and *Drosophila*,^85^ but never in a species that also carries *Galk2*, a paralog of *Galk1*. GALK2 primarily phosphorylates N‐acetylgalactosamine, though in biochemical assays it can phosphorylate Gal when at high concentrations (Km ≈ 4 mM).^83^ Since Gal is elevated in GALT‐deficient mice,^39^ GALK2 in theory could produce some amount of Gal1P. However, given that the *Galt/Galk1* double KO mice showed WT liver and brain levels of Gal1P in this study, GALK2 must not have contributed to the pool of Gal1P detected in these two tissues of the *Galt* ^−/−^ mice. The inability of GALK2 to compensate biochemically in these tissues for the loss of GALK1 is consistent with the poor ability of the *Galk1*‐mutant mouse to metabolize Gal,^30^ and thus GALK2 likely does not produce relevant amounts of Gal1P in the absence of GALK1. However, it is possible that GALK2 contributes to the Gal1P pool in tissues besides liver and brain, or that loss of GALK1 leads to downregulation of GALK2 such that its contribution to the pool of Gal1P is obscured in the *Galt/Galk1* double KO mice.

Normalization of Gal1P levels in the *Galt/Galk1* double KO mouse supports the notion that GALK1 inhibition by a small molecule or other agents should also reduce Gal1P in *Galt*‐deficient mice or humans. This therapeutic strategy is currently being explored^86^ and is based on the observation that GALK1‐deficiency appears to be a less severe human disease relative to GALT‐deficiency.^2^, ^87^, ^88^ Unlike Gal and other Gal metabolites, Gal1P is uniquely elevated in GALT‐deficiency versus GALK1‐deficiency, thus perhaps elevated Gal1P is a driver of pathology in CG.

### 4.2 Modestly reduced cerebrosides and unchanged gangliosides in the *Galt* ^−/−^ mouse

A product of GALT is UDP‐Gal, which is the Gal donor molecule for glycans.^58^ While there are contradictory reports on whether GALT deficiency causes UDP‐Gal‐deficiency,^32^, ^33^, ^34^, ^35^, ^36^, ^37^, ^38^, ^39^, ^40^ there are several reports that glycan structures are Gal‐deficient, reduced, or irregular.^41^, ^42^, ^43^, ^44^, ^45^, ^46^, ^47^, ^48^, ^49^, ^50^, ^51^, ^52^ We explored whether brain glycosphingolipids are deficient in GALT‐deficient mice and found that GalCer 24:1 and sulfatide 24:1 were reduced. This irregularity may be a relevant finding since GalCer and its sulfated derivative make up approximately 25% of myelin,^71^ GalCer 24:1 makes up the greatest fraction of cerebrosides in myelin,^75^ cerebrosides are necessary for stability and maintenance of myelin,^72^, ^73^, ^74^ and an emergent feature of CG is white matter irregularity.^41^, ^60^, ^61^, ^62^, ^63^, ^64^, ^65^, ^66^, ^67^, ^68^, ^69^, ^70^ A limitation of our study is that whole brain was analyzed and it is possible that different regions of the brain are impacted differently. Reductions in GalCer 24:1 and sulfatide 24:1 in the GALT‐deficient mouse brain might occur because UDP‐Gal is reduced. If true, that reduction in UDP‐Gal is likely mild, as gangliosides were not detectably altered. This might suggest that GalCer 24:1 and sulfatide 24:1 are particularly sensitive to changes in UDP‐Gal levels.

GlcCer 24:1 levels were also reduced in the CG mouse brain (Figure 4E,F). UDP‐Glc can also be synthesized from UDP‐Gal via the enzyme GALE (Figure 1). When GALT is deficient, perhaps UDP‐Glc is depleted to generate UDP‐Gal via GALE. This may especially be true in circumstances where the demand for UDP‐Gal is high. Such is the case in 3‐week‐old mouse brains, as this timepoint is a stage in development when GalCer 24:1 levels increase significantly to support myelination,^89^, ^90^ and might explain why the largest effect on GlcCer 24:1 was observed in that age group (Figure 4E).

Individual case studies in patients with CG have reported that cerebrosides were reduced in one CG brain autopsy^41^ and GalCer was reduced in another.^42^ However, to our knowledge, characterization of cerebroside species has not previously been studied in a CG model. While there was variability in the levels of the cerebrosides measured in this mouse model of CG, the 24:1 species of GalCer, which make up the greatest proportion of myelin cerebrosides,^75^ and its sulfated derivative were each reduced by approximately 20% (Figure 4B,D). These observations lend support to the hypothesis that alterations in these 24:1 cerebrosides in CG contribute to pathological changes in white matter, consistent with white matter abnormalities reported in neuroimaging studies of CG patients.^41^, ^60^, ^61^, ^62^, ^63^, ^64^, ^65^, ^66^, ^67^, ^68^, ^69^, ^70^

### 4.3 Conclusions

The results of this study in *Galt* KO mice and *Galt/Galk1* double KO mice provide genetic support that GALK1 is the primary source of elevated Gal1P in CG. If the persistent elevation of Gal1P in patients is a driver of pathology and the long‐term complications in CG, then inhibition of GALK1 provides a potential therapeutic target for investigation. Subtly reduced 24:1 cerebrosides observed in the GALT‐deficient model used in this study may also provide insights into the disease pathology of CG that are worthy of further study.

## AUTHOR CONTRIBUTIONS

BEC and TAH conceptualized the study; LM, RL, MEL, TCG, JR, BEC, and TAH designed the study methodologies; LM, MEL, TCG, CRB, JR, and TAH performed the study investigations; BEC was responsible for study supervision; CRB, HN, and CS conducted the statistical analysis; LM and TAH wrote the original draft of the manuscript; All authors were involved in reviewing and editing the manuscript and approved the final version for submission. TAH is the guarantor of this work and as such, had full access to all the data in the study, takes responsibility for the integrity of the data and the accuracy of the data analysis, and controlled the decision to publish.

## FUNDING INFORMATION

This research was funded by BioMarin Pharmaceutical, Inc. (www.biomarin.com). The funder contributed to the study design, data collection and analysis, decision to publish and preparation of the manuscript, and is represented in the authorship, as described in the “Author contributions.” Editorial assistance provided by FourWave Medical Communications for preparation of manuscript and artwork files was funded by BioMarin Pharmaceutical, Inc.

## CONFLICT OF INTEREST STATEMENT

LM, CRB, CS, BEC, and TAH are employees and shareholders of BioMarin Pharmaceutical, Inc. MEL and HN are employees of BioMarin Pharmaceutical, Inc. TCG is a former employee of and holds shares in BioMarin Pharmaceutical, Inc. RL and JR are former employees of BioMarin Pharmaceutical, Inc.

## PATIENT CONSENT

Not applicable.

## ETHICS STATEMENT

All animal work was performed in strict accordance with the recommendations in the Guide for the Care and Use of Laboratory Animals of the National Institutes of Health under Institutional Animal Care and Use Committee (IACUC)‐reviewed and approved protocols: Buck IACUC committee protocol #A10197 (Buck Institute for Research on Aging, Novato, CA, USA) and IACUC Protocol Number 10010 for Genetic Engineering Technologies (In Vivo Services at The Jackson Laboratory, Sacramento, CA, USA).

## Supporting information


**Figure S1.** Western blot analysis for GALT protein of liver samples from wildtype (WT) and *Galt* knockout (*Galt* ^−/−^) mice.
**Figure S2.** Many non‐24:1 cerebroside species trend toward a modest reduction in the brains of GALT‐deficient mice.
**Table S1.** Gal1P levels in *Galt* knockout (*Galt* ^−/−^), *Galk1* knockout (*Galk1* ^−/−^), and *Galt*/*Galk1* double mutant 3‐week‐old male mice.
**Table S2.** Cerebroside levels in the brain of 3‐week‐old male WT and GALT‐deficient mice.
**Table S3.** Cerebroside levels in the brain of 9‐week‐old male WT and GALT‐deficient mice.
**Table S4.** Cerebroside levels in the brain of 11‐week‐old female WT and GALT‐deficient mice.
**Table S5.** Cerebroside levels in the brain of 39‐week‐old female WT and GALT‐deficient mice.
**Table S6.** Ganglioside levels in the brain of 3‐week‐old male WT and GALT‐deficient mice.
**Table S7.** Ganglioside levels in the brain of 9‐week‐old male WT and GALT‐deficient mice.
**Table S8.** Ganglioside levels in the brain of 11‐week‐old female WT and GALT‐deficient mice.
**Table S9.** Gal1P levels in liver and brain from individual animals included in the analyses: WT, *Galt* KO, *Galk1* KO, and *Galt/Galk1* double mutant 3‐week‐old male mice.
**Table S10.** Levels of cerebrosides in brain samples from individual WT and *Galt* KO animals included in the analyses.
**Table S11.** Levels of gangliosides in brain samples from individual WT and *Galt* KO animals included in the analyses.

## Data Availability

All relevant data are within the manuscript and its Supplementary Materials. In addition to summary data presented in the main text and supplementary tables, the complete raw data set from individual animals included in the analyses are presented in Tables S9–S11 in the Supplementary Materials.
