# Supplementary material for: Galactokinase 1 is the source of elevated galactose‐1‐phosphate and cerebrosides are modestly reduced in a mouse model of classic galactosemia
Source: JIMD Rep. 2024 Jun 23;65(4):280–94. doi: 10.1002/jmd2.12438 (PMC11224506; doi:10.1002/jmd2.12438)
Supplement: Supplementary file 1 — Figure S1. Western blot analysis for GALT protein of liver samples from wildtype (WT) and Galt knockout (Galt −/−) mice. Figure S2. Many non‐24:1 cerebroside species trend toward a modest reduction in the brains of GALT‐deficient mice. Table S1. Gal1P levels in Galt knockout (Galt −/−), Galk1 knockout (Galk1 −/−), and Galt/Galk1 double mutant 3‐week‐old male mice. Table S2. Cerebroside levels in the brain of 3‐week‐old male WT and GALT‐deficient mice. Table S3. Cerebroside levels in the brain of 9‐week‐old male WT and GALT‐deficient mice. Table S4. Cerebroside levels in the brain of 11‐week‐old female WT and GALT‐deficient mice. Table S5. Cerebroside levels in the brain of 39‐week‐old female WT and GALT‐deficient mice. Table S6. Ganglioside levels in the brain of 3‐week‐old male WT and GALT‐deficient mice. Table S7. Ganglioside levels in the brain of 9‐week‐old male WT and GALT‐deficient mice. Table S8. Ganglioside levels in the brain of 11‐week‐old female WT and GALT‐deficient mice. Table S9. Gal1P levels in liver and brain from individual animals included in the analyses: WT, Galt KO, Galk1 KO, and Galt/Galk1 double mutant 3‐week‐old male mice. Table S10. Levels of cerebrosides in brain samples from individual WT and Galt KO animals included in the analyses. Table S11. Levels of gangliosides in brain samples from individual WT and Galt KO animals included in the analyses. [file JMD2-65-280-s001.pdf]

Supplementary Material

Figure S1. Western blot analysis for GALT protein of liver samples from wildtype (WT) and *Galt* knockout (*Galt*$^{-/-}$) mice. 2

Figure S2. Many non-24:1 cerebroside species trend toward a modest reduction in the brains of GALT-deficient mice. 3

Table S1. Gal1P levels in *Galt* knockout (*Galt*$^{-/-}$), *Galk1* knockout (*Galk1*$^{-/-}$), and *Galt/Galk1* double mutant 3-week-old male mice. 4

Table S2. Cerebroside levels in the brain of 3-week-old male WT and GALT-deficient mice. 4

Table S3. Cerebroside levels in the brain of 9-week-old male WT and GALT-deficient mice. 5

Table S4. Cerebroside levels in the brain of 11-week-old female WT and GALT-deficient mice. 5

Table S5. Cerebroside levels in the brain of 39-week-old female WT and GALT-deficient mice. 6

Table S6. Ganglioside levels in the brain of 3-week-old male WT and GALT-deficient mice. 7

Table S7. Ganglioside levels in the brain of 9-week-old male WT and GALT-deficient mice. 7

Table S8. Ganglioside levels in the brain of 11-week-old female WT and GALT-deficient mice. 7

Table S9. Gal1P levels in liver and brain from individual animals included in the analyses: WT, Galt KO, Galk1 KO, and Galt/Galk1 double mutant 3-week-old male mice. 8

Table S10. Levels of cerebrosides in brain samples from individual WT and Galt KO animals included in the analyses. 9

Table S11. Levels of gangliosides in brain samples from individual WT and *Galt* KO animals included in the analyses. 11

Figure S1. Western blot analysis for GALT protein of liver samples from wildtype (WT) and *Galt* knockout (*Galt*$^{-/-}$) mice.

The results confirm that there was no detectable GALT protein in our *Galt* KO mouse model. GALT, galactose-1-phosphate uridylyltransferase.

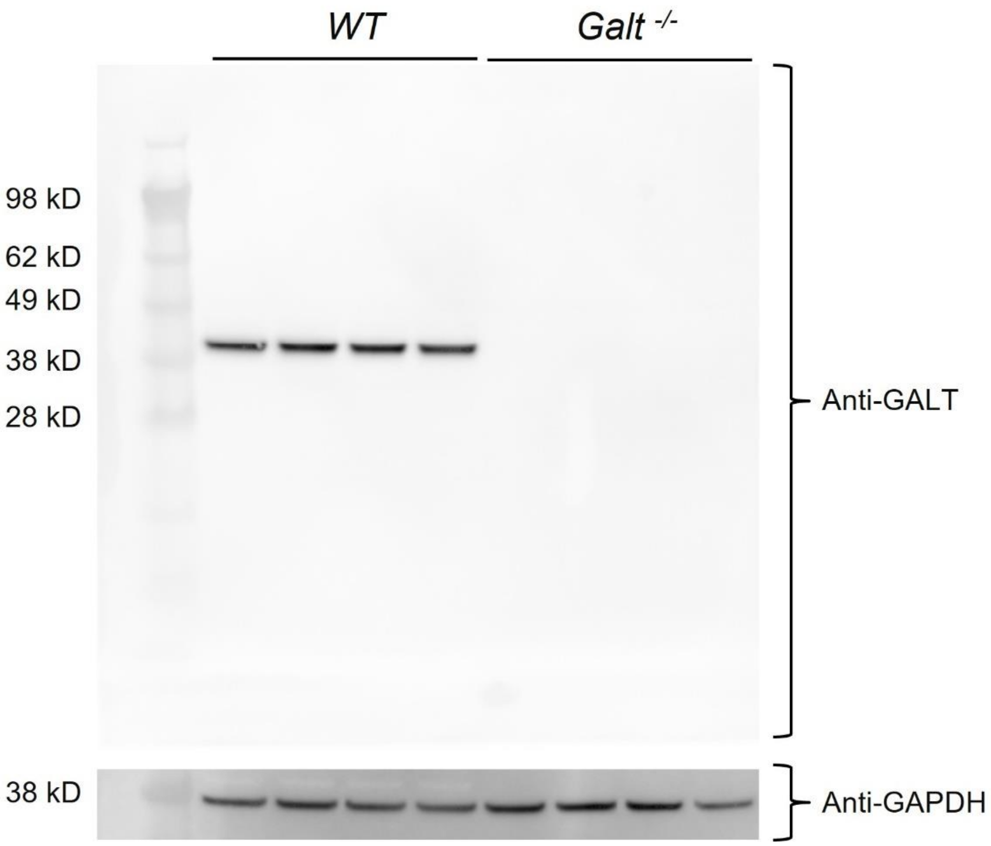

Figure S2. Many non-24:1 cerebroside species trend toward a modest reduction in the brains of GALT-deficient mice. Ten non-24:1 cerebrosides were measured in brain samples from the four age groups of WT and GALT-deficient (*Galt*$^{-/-}$) mice as described above, using LCMS. Cerebroside values from all age groups combined (n = 16 WT, n = 20 *Galt*$^{-/-}$), expressed as a percentage of WT levels (blue dotted line, 100%) showed that 7 of 10 non-24:1 cerebroside species trended toward reduction in the GALT-deficient animals but none met statistical significance. Data shown are mean values ± SD; dots denote data values for all individual animals included in the experiment. GalCer, galactosylceramide; GlcCer, glucosylceramide; SD, standard deviation; WT, wildtype.

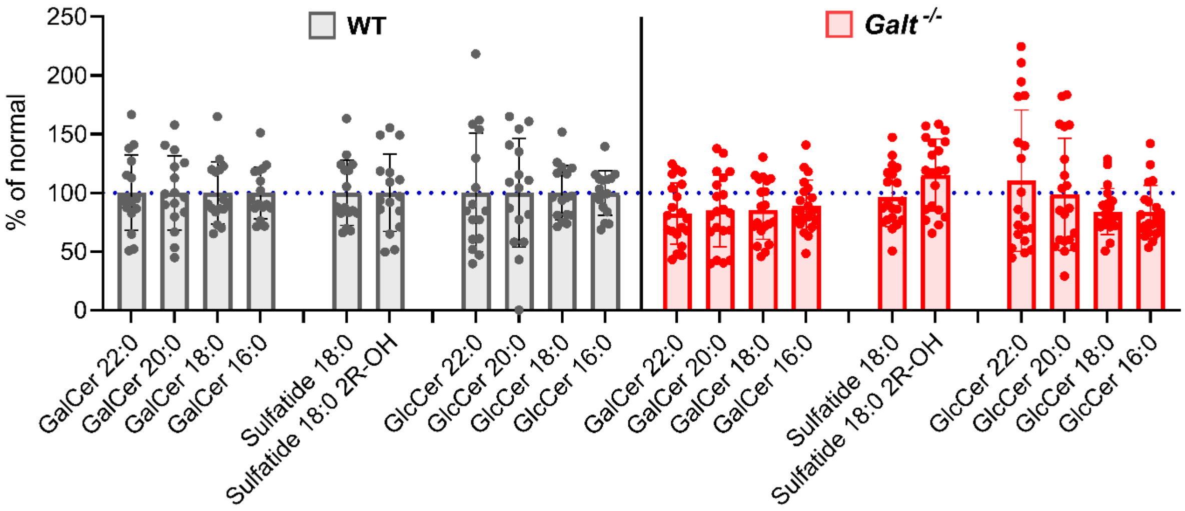

Table S1. Gal1P levels in *Galt* knockout (*Galt*$^{-/-}$), *Galk1* knockout (*Galk1*$^{-/-}$), and *Galt/Galk1* double mutant 3-week-old male mice.

	WT (*N* = 5)	*Galt*$^{-/-}$ (*N* = 5)	*Galk1*$^{-/-}$ (*N* = 5)	*Galt*$^{-/-}$/*Galk1*$^{+/-}$ (*N* = 5)	*Galt*$^{-/-}$/*Galk1*$^{-/-}$ (*N* = 5)
**Liver Gal1P** (ng/mg total protein)	87.6 ± 18.2	1,560.1 ± 282.6	132.2 ± 39.3	1,343.0 ± 369.0	122.6 ± 16.6
**Brain Gal1P** (ng/mg total protein)	37.1 ± 17.4	366.0 ± 66.2	30.9 ± 8.6	305.1 ± 35.9	23.0 ± 5.1

Data shown are mean values ± standard deviation.
Gal1P, galactose-1-phosphate; WT, wildtype.

Table S2. Cerebroside levels in the brain of 3-week-old male WT and GALT-deficient mice.

**Cerebroside measured (pg/µg total protein)**	**Form**	**3-week-old male brain**	
		**WT (*N* = 5)**	***Galt* KO (*N* = 5)**
Galactosylceramide	16:0	10.1 ± 2.5	9.5 ± 2.4
	18:0	180 ± 47	161 ± 32
	20:0	15.0 ± 6.3	13.4 ± 3.4
	22:0	59.4 ± 23.4	49.7 ± 12.4
	24:1	1656 ± 350	1530 ± 262
Glucosylceramide	16:0	1.13 ± 0.13	0.99 ± 0.18
	18:0	25.8 ± 6.4	21.0 ± 3.9
	20:0	0.773 ± 0.243	0.538 ± 0.150
	22:0	1.28 ± 0.60	0.81 ± 0.21
	24:1	11.8 ± 1.1	7.8 ± 0.6
Sulfatide	18:0	88.0 ± 25.2	77.7 ± 15.7
	18:0 2R-OH	7.48 ± 2.91	6.89 ± 2.42
	24:1	308 ± 68	274 ± 38

Data shown are mean values ± standard deviation.
KO, knockout; WT, wildtype.

Table S3. Cerebroside levels in the brain of 9-week-old male WT and GALT-deficient mice.

Cerebroside measured (pg/µg total protein)	Form	9-week-old male brain	
		WT (*N* = 3)	*Galt* KO (*N* = 4)
Galactosylceramide	16:0	13.2 ± 2.1	11.4 ± 1.5
	18:0	178 ± 35	167 ± 29
	20:0	17.4 ± 2.0	16.1 ± 3.6
	22:0	66.0 ± 7.2	61.5 ± 15.7
	24:1	4683 ± 589	3690 ± 398
Glucosylceramide	16:0	1.33 ± 0.30	0.98 ± 0.14
	18:0	17.7 ± 3.6	15.8 ± 1.4
	20:0	0.309 ± 0.272	0.453 ± 0.095
	22:0	0.250 ± 0.136	0.361 ± 0.134
	24:1	7.56 ± 1.27	6.58 ± 0.47
Sulfatide	18:0	133 ± 37	142 ± 28
	18:0 2R-OH	7.58 ± 1.30	9.48 ± 2.28
	24:1	1235 ± 153	975 ± 89

Data shown are mean values ± standard deviation.
KO, knockout; WT, wildtype.

Table S4. Cerebroside levels in the brain of 11-week-old female WT and GALT-deficient mice.

Cerebroside measured (pg/µg total protein)	Form	11-week-old female brain	
		WT (*N* = 4)	*Galt* KO (*N* = 4)
Galactosylceramide	16:0	15.2 ± 2.3	13.3 ± 2.9
	18:0	187 ± 36	173.9 ± 39.4
	20:0	17.5 ± 4.6	19.1 ± 5.3
	22:0	72.2 ± 18.5	67.1 ± 16.4
	24:1	5689 ± 1217	4321 ± 993
Glucosylceramide	16:0	1.45 ± 0.31	1.29 ± 0.36
	18:0	18.8 ± 3.5	17.9 ± 3.2
	20:0	0.359 ± 0.169	0.524 ± 0.147
	22:0	0.234 ± 0.085	0.450 ± 0.087
	24:1	8.48 ± 1.26	7.76 ± 1.34
Sulfatide	18:0	133 ± 25	139 ± 27
	18:0 2R-OH	6.79 ± 2.64	8.30 ± 2.24
	24:1	1432 ± 231	1192 ± 222

Data shown are mean values ± standard deviation.
KO, knockout; WT, wildtype.

Table S5. Cerebroside levels in the brain of 39-week-old female WT and GALT-deficient mice.

Cerebroside measured (pg/µg total protein)	Form	39-week-old female brain	
		**WT (*N* = 4)**	***Galt* KO (*N* = 7)**
Galactosylceramide	16:0	10.8 ± 3.8	9.38 ± 3.26
	18:0	123 ± 54	90 ± 41
	20:0	29.3 ± 12.7	18.5 ± 8.9
	22:0	71.3 ± 34.6	49.3 ± 21.9
	24:1	2645 ± 1077	1942 ± 958
Glucosylceramide	16:0	0.735 ± 0.208	0.609 ± 0.233
	18:0	15.1 ± 5.4	11.5 ± 3.8
	20:0	0.643 ± 0.289	0.365 ± 0.109
	22:0	0.435 ± 0.356	0.338 ± 0.126
	24:1	3.56 ± 1.40	2.82 ± 1.17
Sulfatide	18:0	58.3 ± 25.2	52.8 ± 19.2
	18:0 2R-OH	4.00 ± 1.65	4.89 ± 1.06
	24:1	1334 ± 496	1117 ± 450

Data shown are mean values ± standard deviation.
KO, knockout; WT, wildtype.

Table S6. Ganglioside levels in the brain of 3-week-old male WT and GALT-deficient mice.

Ganglioside measured (ng/µg total protein)	3-week-old male brain	
	WT	*Galt* KO
GM1	2.61 ± 0.26	2.68 ± 0.22
GM2	0.0576 ± 0.0043	0.0580 ± 0.0062
GM3	0.121 ± 0.008	0.131 ± 0.009
GA1	0.0491 ± 0.0060	0.0441 ± 0.0025
GA2*	ND	ND

*GA2 levels undetectable (ND) at 21 days
Data shown are mean values ± standard deviation.
N = 5 per group.
KO, knockout; WT, wildtype.

Table S7. Ganglioside levels in the brain of 9-week-old male WT and GALT-deficient mice.

Ganglioside measured (ng/µg total protein)	9-week-old male brain	
	WT (*N* = 3)	*Galt* KO (*N* = 4)
GM1	2.59 ± 0.34	2.51 ± 0.32
GM2	0.095 ± 0.007	0.094 ± 0.014
GM3	0.232 ± 0.020	0.276 ± 0.073
GA1	0.044 ± 0.008	0.037 ± 0.007
GA2	0.010 ± 0.002	0.008 ± 0.006

Data shown are mean values ± standard deviation.
KO, knockout; WT, wildtype.

Table S8. Ganglioside levels in the brain of 11-week-old female WT and GALT-deficient mice.

Ganglioside measured (ng/µg total protein)	11-week-old female brain	
	WT (*N* = 4)	*Galt* KO (*N* = 4)
GM1	3.55 ± 0.45	3.06 ± 0.26
GM2	0.108 ± 0.012	0.091 ± 0.013
GM3	0.314 ± 0.036	0.310 ± 0.078
GA1	0.048 ± 0.002	0.043 ± 0.006
GA2	0.017 ± 0.002	0.014 ± 0.002

Data shown are mean values ± standard deviation.
KO, knockout; WT, wildtype.

Table S9. Gal1P levels in liver and brain from individual animals included in the analyses: WT, Galt KO, Galk1 KO, and Galt/Galk1 double mutant 3-week-old male mice.
Gal1P, galactose-1-phosphate; KO, knockout; WT, wildtype.

**Gal1P (ng/mg total protein) in LIVER from 3-week-old male mice**					
WT					
**Mouse ID#**	**1**	**2**	**3**	**4**	**5**
Gal1P	103.4992525	106.4528219	88.37641385	76.99815182	62.89763026
Galt $^{-/-}$					
**Mouse ID#**	**6**	**7**	**8**	**9**	**10**
Gal1P	1326.231324	1453.88033	1447.77688	2049.364714	1523.018707
Galk1 $^{-/-}$					
**Mouse ID#**	**11**	**12**	**13**	**14**	**15**
Gal1P	196.5874393	99.98260228	138.4409151	123.1541526	102.8549719
Galt $^{-/-}$; ***Galk1*** $^{+/-}$					
**Mouse ID#**	**16**	**17**	**18**	**19**	**20**
Gal1P	1010.957634	1522.001131	1177.742595	1904.965224	1099.480885
Galt $^{-/-}$; ***Galk1*** $^{-/-}$					
**Mouse ID#**	**21**	**22**	**23**	**24**	**25**
Gal1P	142.649306	122.8616559	104.3145781	135.1159778	108.0268884

**Gal1P (ng/mg total protein) in BRAIN from 3-week-old male mice**					
WT					
**Mouse ID#**	**1**	**2**	**3**	**4**	**5**
Gal1P	22.91711651	66.77901549	37.38403776	27.91192758	30.26294401
Galt $^{-/-}$					
**Mouse ID#**	**6**	**7**	**8**	**9**	**10**
Gal1P	412.9439784	302.6795209	376.6678731	443.5676033	293.9501692
Galk1 $^{-/-}$					
**Mouse ID#**	**11**	**12**	**13**	**14**	**15**
Gal1P	45.90097544	28.28557703	29.2370569	24.4966274	26.37421287
Galt $^{-/-}$; ***Galk1*** $^{+/-}$					
**Mouse ID#**	**16**	**17**	**18**	**19**	**20**
Gal1P	265.6556458	322.0711563	350.3867147	271.2870997	316.1477119
Galt $^{-/-}$; ***Galk1*** $^{-/-}$					
**Mouse ID#**	**21**	**22**	**23**	**24**	**25**
Gal1P	23.38503058	20.34621654	23.34324948	30.80719564	17.14858612

Table S10. Levels of cerebrosides in brain samples from individual WT and Galt KO animals included in the analyses.
GalCer, galactosylceramide; GlcCer, glucosylceramide; KO, knockout; WT, wildtype

Cerebrosides (pg/µg total protein) in BRAIN from 3-week-old male mice												
	WT					*Galt* $^{-/-}$						
**Mouse ID#**	**1**	**2**	**3**	**4**	**5**	**6**	**7**	**8**	**9**	**10**	**NA**	**NA**
GalCer 24:1	2081.313	1768.58	1840.028	1329.855	1261.595	1615.94	1886.178	1601.773	1244.848	1300.248	NA	NA
GalCer 22:0	68.3575	75.4475	83.7425	30.9775	38.3625	69.9275	51.9775	46.2975	38.485	41.8875	NA	NA
GalCer 20:0	18.3625	18.8725	21.125	6.725	10.0175	17.7375	16.03	12.6425	10.175	10.24	NA	NA
GalCer 18:0	215.0925	211.6125	214.725	130.3725	125.995	203.7125	180.715	158.9	128.4675	134.65	NA	NA
GalCer 16:0	11.0675	11.9375	12.4325	7.6525	7.165	12.24	11.8625	8.42	7.12	7.735	NA	NA
Sulfatide 24:1	395.735	328.805	336.0025	252.19	228.705	286.74	326.27	282.345	237.6375	235.3625	NA	NA
Sulfatide 18:0	109.07	104.9425	105.1225	61.3825	59.5575	101.8625	81.9575	75.865	61.09	67.5875	NA	NA
Sulfatide 18:0 2R-OH	8.7725	8.3125	11.155	5.31	3.8475	11.015	6.4175	6.6525	4.91	5.4325	NA	NA
GlcCer 24:1	12.3825	13.045	12.23	11.285	10.1325	7.9075	8.34	7.665	6.9	8.4325	NA	NA
GlcCer 22:0	1.3375	1.66	2.03	0.6025	0.7725	1.1	0.6575	0.8925	0.5725	0.8275	NA	NA
GlcCer 20:0	0.855	1.0875	0.8425	0.63	0.4475	0.695	0.3925	0.68	0.535	0.385	NA	NA
GlcCer 18:0	31.2375	30.14	29.9625	19.33	18.4025	27.6475	18.5475	21.085	19.24	18.335	NA	NA
GlcCer 16:0	1.1175	1.065	1.2575	0.945	1.2625	1.2325	0.92	0.99	0.745	1.065	NA	NA

Cerebrosides (pg/µg total protein) in BRAIN from 9-week-old male mice												
	WT					*Galt* $^{-/-}$						
**Mouse ID#**	**26**	**27**	**28**	**NA**	**NA**	**29**	**30**	**31**	**32**	**NA**	**NA**	**NA**
GalCer 24:1	4187.623	4529.193	5334.843	NA	NA	3514.62	3915.845	3222.143	4107.128	NA	NA	NA
GalCer 22:0	62.61	61.0625	74.315	NA	NA	63.78	82.1875	55.0425	45.1575	NA	NA	NA
GalCer 20:0	15.83	16.7125	19.5675	NA	NA	17.105	20.54	14.3825	12.27	NA	NA	NA
GalCer 18:0	154.545	160.34	217.735	NA	NA	179.38	198.965	158.9225	130.13	NA	NA	NA
GalCer 16:0	11.5825	12.425	15.5875	NA	NA	10.13	11.7375	10.47	13.345	NA	NA	NA
Sulfatide 24:1	1105.978	1193.91	1404.175	NA	NA	875.0675	1036.4	925.9175	1063.658	NA	NA	NA
Sulfatide 18:0	112.85	110.37	176.2275	NA	NA	144.68	164.775	155.9075	102.6325	NA	NA	NA
Sulfatide 18:0 2R-OH	6.4175	7.345	8.975	NA	NA	9.04	11.6025	10.82	6.475	NA	NA	NA
GlcCer 24:1	6.9375	6.735	9.02	NA	NA	5.9275	6.575	6.8625	6.97	NA	NA	NA
GlcCer 22:0	0.1925	0.1525	0.405	NA	NA	0.3575	0.455	0.4575	0.1725	NA	NA	NA
GlcCer 20:0	0	0.51	0.4175	NA	NA	0.355	0.5675	0.49	0.3975	NA	NA	NA
GlcCer 18:0	17.03	14.43	21.5575	NA	NA	15.4475	17.615	16.0225	14.165	NA	NA	NA
GlcCer 16:0	1.54	0.985	1.4725	NA	NA	0.78	1.0375	1.1075	0.975	NA	NA	NA

Table S10. *Continued.* GalCer, galactosylceramide; GlcCer, glucosylceramide; WT, wildtype

Cerebrosides (pg/µg total protein) in BRAIN from 11-week-old female mice												
	WT					*Galt* $^{-/-}$						
**Mouse ID#**	**33**	**34**	**35**	**36**	**NA**	**37**	**38**	**39**	**40**	**NA**	**NA**	**NA**
GalCer 24:1	5317.6525	4879.56	7494.5875	5064.1525	NA	5267.9925	3466.28	3461.6475	5089.77	NA	NA	NA
GalCer 22:0	62.5675	59.6325	99.62	67.04	NA	77.4675	48.665	58.265	83.8375	NA	NA	NA
GalCer 20:0	13.9225	14.5675	23.88	17.4875	NA	24.0525	13.98	15.055	23.3775	NA	NA	NA
GalCer 18:0	161.81	168.885	240.4125	176.0325	NA	208.8825	133.465	138.9925	214.3125	NA	NA	NA
GalCer 16:0	15.515	13.2525	18.3975	13.765	NA	16.595	12.805	9.62	14.245	NA	NA	NA
Sulfatide 24:1	1313.565	1281.8425	1774.825	1355.93	NA	1396.03	1083.04	931.9075	1357.235	NA	NA	NA
Sulfatide 18:0	109.4675	116.38	164.6525	139.7525	NA	144.915	119.6075	115.1375	174.745	NA	NA	NA
Sulfatide 18:0 2R-OH	6.365	4.4025	10.5475	5.8375	NA	8.0875	8.965	5.39	10.7575	NA	NA	NA
GlcCer 24:1	8.81	8.825	9.6125	6.67	NA	7.9125	8.1125	5.9075	9.115	NA	NA	NA
GlcCer 22:0	0.1975	0.36	0.18	0.1975	NA	0.525	0.3275	0.4925	0.455	NA	NA	NA
GlcCer 20:0	0.155	0.3125	0.555	0.415	NA	0.3125	0.5675	0.5625	0.655	NA	NA	NA
GlcCer 18:0	15.185	18.3025	23.63	18.0325	NA	17.4725	16.785	13.99	23.3275	NA	NA	NA
GlcCer 16:0	1.6775	1.4925	1.6225	0.9925	NA	1.0325	1.3375	1.0125	1.7925	NA	NA	NA

Cerebrosides (pg/µg total protein) in BRAIN from 39-week-old female mice												
	WT					*Galt* $^{-/-}$						
**Mouse ID#**	**41**	**42**	**43**	**44**	**NA**	**45**	**46**	**47**	**48**	**49**	**50**	**51**
GalCer 24:1	2333.645	2158.14	4233.098	1855.12	NA	3005.043	1176.843	3596.238	1350.115	1330.845	1414.533	1723.73
GalCer 22:0	67.9525	62.28	118.885	36.1925	NA	73.9925	30.72	85.5325	34.1075	33.285	38.735	48.3575
GalCer 20:0	29.0525	26.3275	46.2475	15.5825	NA	28.42	12.455	33.0675	12.4325	11.635	11.7825	19.62
GalCer 18:0	105.665	102.7425	202.445	80.0175	NA	135.3225	56.19	160.0225	66.57	60.53	68.4525	83.21
GalCer 16:0	9.435	9.7575	16.3575	7.7675	NA	11.2525	5.22	15.2375	7.955	7.1575	8.475	10.3775
Sulfatide 24:1	1192.928	1170.343	2054.84	918.9675	NA	1582.953	641.9275	1891.113	855.37	863.2725	931.3825	1052.978
Sulfatide 18:0	50.9675	48.6025	95.27	38.52	NA	70.89	29.3725	85.95	42.5625	43.7375	46.045	50.7175
Sulfatide 18:0 2R-OH	3.68	4.3725	5.965	1.98	NA	4.69	3.065	6.28	4.7425	4.2475	5.74	5.435
GlcCer 24:1	3.4	2.5075	5.575	2.745	NA	3.76	1.6225	5.005	2.34	2.1375	2.145	2.75
GlcCer 22:0	0.3925	0.225	0.95	0.1725	NA	0.2575	0.2125	0.5625	0.23	0.315	0.4375	0.3475
GlcCer 20:0	0.67	0.495	1.035	0.3725	NA	0.4225	0.1875	0.5475	0.3475	0.3225	0.345	0.385
GlcCer 18:0	14.155	11.1425	22.925	12.21	NA	11.21	7.63	19.435	10.72	8.635	11.0375	11.7575
GlcCer 16:0	0.73	0.645	1.025	0.54	NA	0.81	0.3925	1.045	0.48	0.5275	0.4625	0.5425

Table S11. Levels of gangliosides in brain samples from individual WT and *Galt* KO animals included in the analyses.

KO, knockout; WT, wildtype

**Gangliosides (ng/µg total protein) in BRAIN from 3-week-old male mice**										
	WT					***Galt*** $^{-/-}$				
**Mouse ID#**	**1**	**2**	**3**	**4**	**5**	**6**	**7**	**8**	**9**	**10**
GM1	2.7951	2.61107	2.576585	2.873558	2.211578	2.720905	2.763778	2.522768	2.967278	2.404518
GM2	0.063993	0.059324	0.052994	0.055001	0.056539	0.059972	0.058366	0.066701	0.054828	0.049963
GM3	0.12948	0.128549	0.121243	0.116912	0.110059	0.140906	0.134483	0.118784	0.136041	0.125799
GA1	0.0524	0.042219	0.04598	0.057629	0.047087	0.044077	0.042536	0.045257	0.047461	0.041018
GA2	ND	ND	ND	ND	ND	ND	ND	ND	ND	ND

**Gangliosides (ng/µg total protein) in BRAIN from 9-week-old male mice**										
	WT					***Galt*** $^{-/-}$				
**Mouse ID#**	**26**	**27**	**28**	**NA**	**NA**	**29**	**30**	**31**	**32**	**NA**
GM1	2.244	2.926	2.585	NA	NA	2.2385	2.277	2.5905	2.9315	NA
GM2	0.087175	0.10175	0.096938	NA	NA	0.074525	0.095425	0.097488	0.106838	NA
GM3	0.2134	0.25355	0.2299	NA	NA	0.2024	0.23155	0.30855	0.36245	NA
GA1	0.036438	0.0528	0.043725	NA	NA	0.02805	0.03685	0.039738	0.044275	NA
GA2	0.008525	0.011275	0.011688	NA	NA	0	0.010038	0.010313	0.013063	NA

**Gangliosides (ng/µg total protein) in BRAIN from 11-week-old female mice**										
	WT					***Galt*** $^{-/-}$				
**Mouse ID#**	**33**	**34**	**35**	**36**	**NA**	**37**	**38**	**39**	**40**	**NA**
GM1	3.7895	2.97	3.9985	3.421	NA	2.7885	3.3275	2.893	3.2395	NA
GM2	0.117975	0.092538	0.117563	0.102025	NA	0.078925	0.08965	0.085938	0.109725	NA
GM3	0.3421	0.28105	0.34815	0.286	NA	0.2244	0.4037	0.2739	0.33825	NA
GA1	0.05005	0.044963	0.0495	0.046475	NA	0.037125	0.044688	0.039325	0.049638	NA
GA2	0.0187	0.014575	0.018838	0.015675	NA	0.011413	0.013475	0.013613	0.016775	NA
